# Tumor microenvironments with an active type I IFN response are sensitive to inhibitors of heme degradation

**DOI:** 10.1172/jci.insight.191017

**Published:** 2025-07-08

**Authors:** Dominika Sosnowska, Tik Shing Cheung, Jit Sarkar, James W. Opzoomer, Karen T. Feehan, Joanne E. Anstee, Chloé A. Woodman, Mohamed Reda Keddar, Kalum Clayton, Samira Ali, William Macmorland, Dorothy D. Yang, James Rosekilly, Cheryl E. Gillett, Francesca D. Ciccarelli, Richard Buus, James Spicer, Anita Grigoriadis, James N. Arnold

**Affiliations:** 1 School of Cancer and Pharmaceutical Sciences, King’s College London, London, United Kingdom.; 2 Cancer Systems Biology Laboratory, The Francis Crick Institute, London, United Kingdom.; 3 Centre for Cancer Genomics and Computational Biology, Bart’s Cancer Institute, Queen Mary University London, London, United Kingdom.; 4 The Breast Cancer Now Toby Robins Research Centre at The Institute of Cancer Research, London, United Kingdom.; 5 Ralph Lauren Centre for Breast Cancer Research, Royal Marsden Hospital, Fulham Road, London, United Kingdom.

**Keywords:** Immunology, Oncology, Breast cancer, Cancer immunotherapy, Immunotherapy

## Abstract

The tumor microenvironment (TME) is highly heterogeneous and can dictate the success of therapeutic interventions. Identifying TMEs that are susceptible to specific therapeutic interventions paves the way for more personalized and effective treatments. In this study, using a spontaneous murine model of breast cancer, we characterize a TME that is responsive to inhibitors of the heme degradation pathway mediated by heme oxygenase (HO), resulting in CD8^+^ T cell– and NK cell–dependent tumor control. A hallmark of this TME is a chronic type I interferon (IFN) signal that is directly involved in orchestrating the antitumor immune response. Importantly, we identify that similar TMEs exist in human breast cancer that are associated with patient prognosis. Leveraging these observations, we demonstrate that combining a STING agonist, which induces type I IFN responses, with an HO inhibitor produces a synergistic effect leading to superior tumor control. This study highlights HO activity as a potential resistance mechanism for type I IFN responses in cancer, supporting a therapeutic rationale for targeting the heme degradation pathway to enhance the efficacy of STING agonists.

## Abstract 2

This study identifies a breast tumor microenvironment with chronic type-I IFN signaling, responsive to HO inhibition, uncovering therapeutic synergy between STING agonists and HO inhibitors.

## Graphical abstract

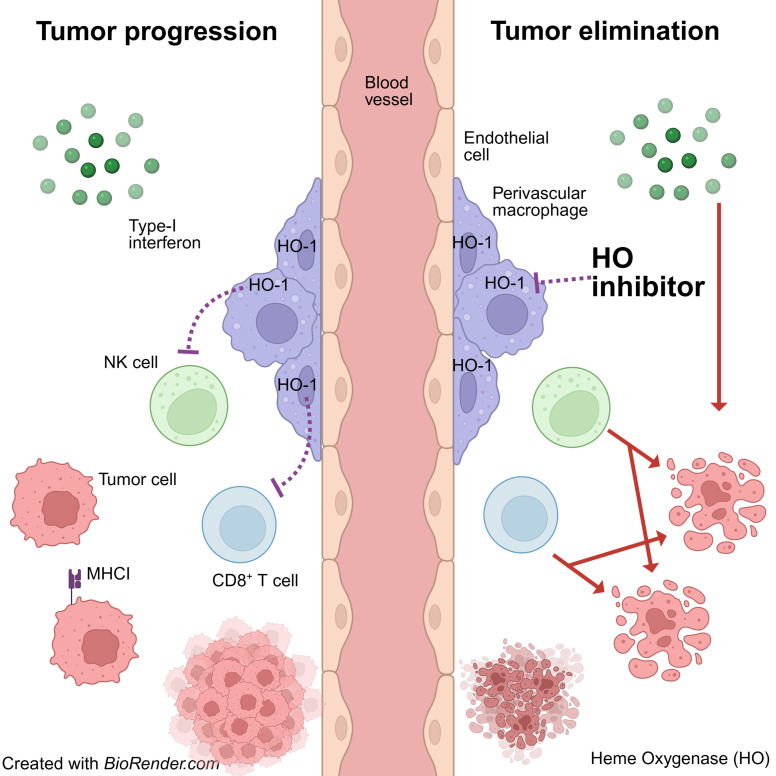

## Introduction

The tumor microenvironment (TME) represents a heterogeneous and dynamic interplay between cancer cells and resident and infiltrating immune and nonimmune cells, extracellular matrix, and soluble and physical factors that can dictate the patient’s treatment outcomes and prognosis (1–5). Immunotherapies, such as those that target the immune checkpoint molecules PD-1 and CTLA-4, have achieved durable remissions in some patients with advanced malignancies, while a considerable number of patients remain refractory to these treatments (6). Such diverse extremes of treatment outcomes highlight the need for a more personalized treatment approach. Decoding TMEs that are responsive to specific therapeutic modalities can lead to improved outcomes. Furthermore, such insight will inform rational approaches to drug combinations, enabling the modulation of TMEs into a more responsive state (7, 8).

The heme degradation pathway in cancer is an emerging immunotherapy target (3, 9, 10). Heme is degraded by the heme oxygenase (HO) family of enzymes, where in humans and mice there are 2 isoforms: HO-1, which is induced in response to stress stimuli, and HO-2, which is constitutively expressed, particularly in the central nervous system (9). Enzymatically active HO-1 is associated with the smooth endoplasmic reticulum and breaks down heme to the biologically active catabolites biliverdin, ferrous iron (Fe^2+^), and carbon monoxide (CO) (9). HO-1 plays a variety of important protumoral roles within the TME, facilitating metastasis (10, 11), tumor cell survival (12–14), and immunosuppression (3, 15). However, HO-targeting drugs rarely deliver tumor control in preclinical models as single agents (11, 15), suggesting that an optimal TME is necessary to observe therapeutic effects (16).

We recently demonstrated that a subset of perivascular tumor-associated macrophages (PvTAMs) exclusively express HO-1 and coexpress lymphatic vessel endothelial hyaluronan receptor-1 (LYVE-1) in *MMTV-PyMT* tumors (3, 11, 17). PvTAMs are characterized by their close spatial association with blood vessels (<15–20 μm) (18, 19). We demonstrated that LYVE-1^+^ PvTAMs are polarized by interleukin 6 (IL-6) secreted by nearby endothelial cells, which also drives expression of the chemokine receptor CCR5 and establishes a communication axis that supports the formation of collaborative multicellular nests of LYVE-1^+^ TAMs within the perivascular niche (3). LYVE-1^+^ TAM expression of HO-1 is biologically important for this TAM subset, as HO-1 activity has been shown to contribute to immune exclusion of CD8^+^ T cells from the TME (3, 15, 20).

The current study explores the immunomodulatory role of HO-1 in cancer and investigates a TME that is responsive to an HO inhibitor. We uncover a TME with a chronic type I interferon (IFN) response that plays a direct role in the tumor control under HO inhibition. We present evidence that TMEs with an active type I IFN response exist in human breast cancer, and that expression of the gene for HO-1 (*HMOX1*) within these tumors is associated with prognosis. We provide preclinical evidence that a TME with either an endogenous or drug-induced type I IFN response may benefit from an HO inhibitor to deliver superior antitumor efficacy.

## Results

### MMTV-PyMT tumors grown in Prf1^–/–^ mice depend on HO activity to sustain their growth.

**Figure 1:**
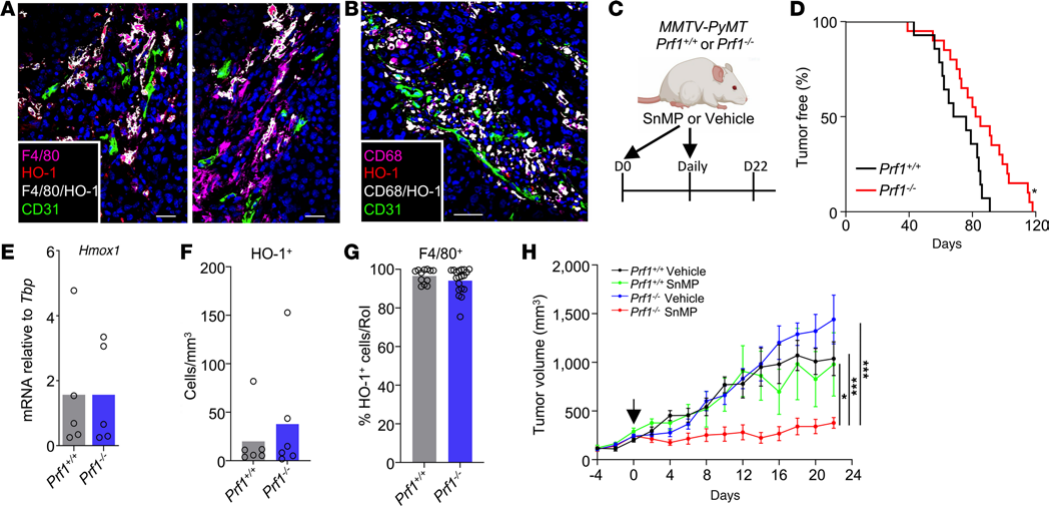
HO activity sustains tumor growth in perforin null *MMTV-PyMT* mice. (**A**) Representative images of frozen sections of a *Prf1^+/+^* *MMTV-PyMT* tumor stained with DAPI (nuclei, blue) and antibodies against CD31 (green), F4/80 (magenta), HO-1 (red), and F4/80 and HO-1 colocalization (white). Scale bars: 25 μm. (**B**) Representative image of a frozen section from human invasive ductal mammary carcinoma stained with DAPI (nuclei, blue) and antibodies against CD31 (green), CD68 (magenta), and HO-1 (red), and CD68 and HO-1 colocalization (white). Scale bar: 50 μm. (**C**) Schematic representing the dosing strategy for SnMP or vehicle in *Prf1^+/+^* or *Prf1^–/–^* *MMTV-PyMT* mice. Mice were treated with SnMP (25 μmol/kg/ daily) or vehicle starting on day 0. (**D**) Kaplan-Meier plot showing the fraction of tumor-free *Prf1^+/+^* (*n* = 28) or *Prf1^–/–^* (*n* = 36) *MMTV-PyMT* mice days after birth. Survival curves were compared using the log-rank (Mantel-Cox) test. (**E**) mRNA expression of *Hmox1* in tumor tissue from *Prf1^+/+^* or *Prf1^–/–^* *MMTV-PyMT* mice treated with vehicle relative to the housekeeping gene *Tbp* (*n* = 5), as assessed using qRT-PCR analysis. (**F** and **G**) Quantitation of HO-1–expressing cells per mm^3^ from frozen *MMTV-PyMT* tumor sections stained with DAPI to mark nuclei and HO-1 (*n* = 6 tumors) (**F**) and the localization of HO-1 with F4/80 across 2–3 sections per tumor (each dot represents a section) (**G**) assessed using immunofluorescence. (**H**) Growth curves of established tumors in *Prf1^+/+^* or *Prf1^–/–^* *MMTV-PyMT* mice treated with SnMP (25 μmol/kg daily) or vehicle (cohorts *n* = 6–8 mice). Panel **C** was created using BioRender software. Statistical analysis of tumor growth curves was performed using the “CompareGrowthCurves” function of the statmod software package. For bar charts, a 2-sided unpaired Students *t* test was used for parametric data, or a Mann-Whitney test for nonparametric data. Bar charts show the mean and the dots show individual data points from individual tumors and mice (and sections in panel **G**). Line charts display the mean and SEM. *P < 0.05, ***P < 0.001.

In the murine *MMTV-PyMT* model of breast cancer, HO-1 is exclusively expressed by PyTAMs (3, 11, 17) (Figure 1A), and a similar macrophage subset is observed in human breast adenocarcinomas (Figure 1B) (3). To investigate the interplay between HO-1 activity in PvTAMs and the antitumor immune response, we crossed *MMTV-PyMT* mice onto a perforin-deficient (*Prf1^–/–^*) background (Figure 1C), thereby impairing cytolytic immune function within the TME as a control for ongoing experiments. Perforin is a pore-forming protein essential to the perforin/granzyme cytolytic pathway, which mediates target cell killing by cytotoxic T and natural killer (NK) cells (21), and plays a key role in immune surveillance of malignant cells (22, 23).

Unexpectedly, loss of perforin delayed tumor onset, with median latency increasing from 72 to 84 days (Figure 1D), a finding that contrasts with the expected acceleration of tumor growth typically observed in immunocompromised mice (24, 25). Analysis of tumor tissues revealed no significant changes in *Hmox1* expression (Figure 1E) or the frequency of HO-1^+^ TAMs (Figure 1F). In both *Prf1^–/–^* and *Prf1^+/+^* tumors, greater than 94% of HO-1^+^ cells colocalized with F4/80, highlighting TAMs to be the major source of HO-1 in both TMEs (Figure 1G). Pharmacologic inhibition of HO activity using tin mesoporphyrin (SnMP) (15, 16, 26) did not affect tumor growth in immunocompetent *MMTV-PyMT* mice (15) (Figure 1H). However, SnMP treatment in *Prf1^–/–^* *MMTV-PyMT* mice resulted in a cessation of tumor growth (Figure 1H). This unexpected result suggests that in the absence of perforin-mediated cytotoxicity, the TME in this model had become reliant on HO activity and the heme degradation pathway to support tumor growth. These findings prompted the need for deeper investigation into the biological basis of this dependency, which could help define the key attributes of a TME responsive to HO inhibitors.

### CD8^+^ T cells and NK cells mediate perforin-independent tumor control following HO inhibition.

**Figure 2:**
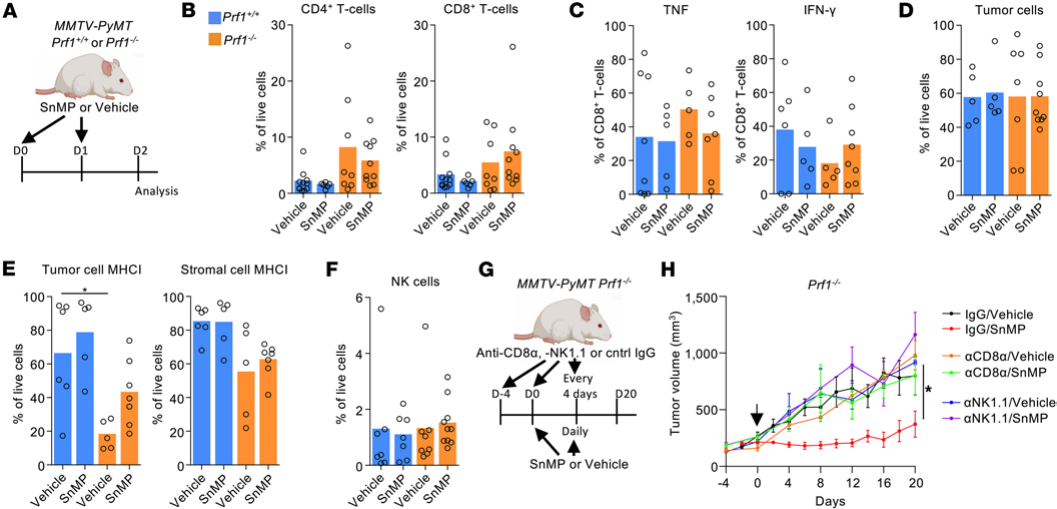
HO activity protects tumors from perforin-independent immunological control of tumor growth. (**A**–**F**) Schematic representing the acute dosing strategy for SnMP or respective vehicle in *Prf1^+/+^* or *Prf1^–/–^* *MMTV-PyMT* mice. Mice were treated with SnMP (25 μmol/kg/ daily) or vehicle for 36 hours starting on day 0 (**A**). Thirty-six hours after initiation of treatment, tumors were harvested, enzyme-dispersed, and cell populations analyzed by flow cytometry for the frequency of CD4^+^ and CD8^+^ T cells (**B**) and their expression of the cytokines TNF and IFN-γ (**C**), tumor cells (**D**), expression of MHCI haplotypes H-2Kb, H-2Db, H-2Kq, and H-2D/Lq on the tumor cells and stroma (**E**), and frequency of NK cells (**F**) (cohorts of *n* = 5–10 mice). (**G** and **H**) Schematic representing the dosing strategy for SnMP or respective vehicle in *Prf1^+/+^* or *Prf1^–/–^* *MMTV-PyMT* mice. Mice were treated with SnMP (25 μmol/kg/ daily) or vehicle and immune-depleting antibodies anti-CD8α, anti-NK1.1 or control IgG (**G**). Growth curves of established tumors in *Prf1^+/+^* or *Prf1^–/–^* *MMTV-PyMT* mice treated with SnMP or vehicle, with anti-CD8α, anti-NK1.1 antibody, or IgG (cohorts of *n* = 5–7 mice) (**H**). Panels **A** and **G** were created using BioRender software. Statistical analysis of tumor growth curves was performed using the “CompareGrowthCurves” function of the statmod software package. For bar charts statistical significance was determined using a Kruskal-Wallis test followed by Dunn’s multiple-comparison test. Bar charts show the mean and the dots show individual data points from individual tumors and mice. Line charts display the mean and SEM. *P < 0.05.

To investigate the mechanism underlying tumor control in *Prf1^–/–^* *MMTV-PyMT* mice treated with the HO inhibitor SnMP, we analyzed tumors 36 hours after the initiation of treatment to capture the earliest biological changes in the TME (Figure 2A). The stromal composition in enzyme-dispersed tumors was assessed using flow cytometry (Supplemental Figure 1; supplemental material available online with this article; https://doi.org/10.1172/jci.insight.191017DS1). Overall, the stromal composition of the tumors was similar between groups, despite the spontaneous nature of the tumor model (Supplemental Figure 2, A–C). SnMP treatment had minimal impact on the prevalence of the major stromal populations, including LYVE-1^+^ TAMs (CD206^hi^MHCII^lo^) and other TAM subsets defined by their CD206 or MHCII expression (Supplemental Figure 2D) (27). Although not significant, there was a tendency toward more CD4^+^ and CD8^+^ T cells in *Prf1^–/–^* tumors relative to *Prf1^+/+^* counterparts (Figure 2B). No change was observed in the expression of Cxcr3-dependent T cell chemokines *Cxcl9*, *Cxcl10*, and *Cxcl11* across conditions (Supplemental Figure 2E). Also, SnMP did not significantly alter T cell infiltration compared to vehicle treatment (Figure 2B), nor did it increase the frequency of TNF- or IFN-γ–producing T cells (Figure 2C and Supplemental Figure 2F). Given perforin’s role in immune editing (22, 23, 28), we next assessed MHCI expression. Interestingly, tumor cells of *Prf1^–/–^* mice exhibited reduced MHCI surface expression compared with those from *Prf1^+/+^* mice (Figure 2E and Supplemental Figure 2G). This downregulation was specific to tumor cells, as stromal cells maintained high MHCI expression (Figure 2E). Loss of MHCI is a known mechanism of immune evasion (29), potentially enabling escape from T cell receptor–mediated noncytolytic control in the absence of perforin (30), but concurrently rendering tumor cells more susceptible to NK cell–mediated killing via “missing-self” recognition (31).

Despite the MHCI loss, NK cell abundance and subset distribution were equivalent between *Prf1^+/+^* and *Prf1^–/–^* tumors (Figure 2F and Supplemental Figure 2H). NK T cells were exceedingly rare in these tumors (typically constituting less than 0.07% of total live cells; data not shown). To investigate a potential functional role of CD8^+^ T cells and NK cells in the tumor control observed in *Prf1^–/–^* *MMTV-PyMT* mice, we performed in vivo depletion of these cell populations prior to SnMP treatment (Figure 2G and Supplemental Figure 3, A–E). In both cases, tumor control was abrogated (Figure 2H), demonstrating that CD8^+^ T cells and NK cells are both essential for the observed antitumor response and that their activity does not rely on perforin-mediated cytotoxicity.

### MMTV-PyMT tumors in Prf1^–/–^ mice display a chronic type I IFN response.

**Figure 3:**
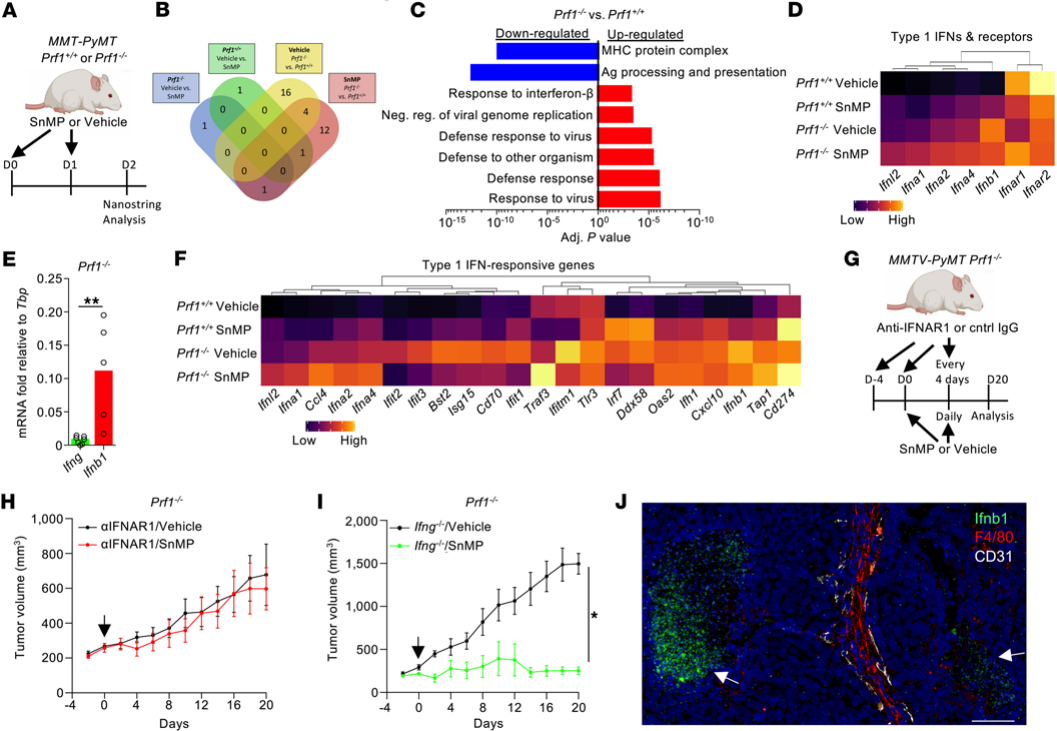
HO inhibition facilitates type I IFN–dependent immunological control of tumor growth. (**A**–**F**) Schematic representing the acute dosing strategy for SnMP or respective vehicle in *Prf1^+/+^* or *Prf1^–/–^* *MMTV-PyMT* mice. Mice were treated with SnMP (25 μmol/kg/ daily) or vehicle for 36 hours starting on day 0 (*n* = 3) (**A**). Venn diagram showing the number of DEGs identified between the compared experimental cohorts (**B**). Gene Ontology (GO) analysis showing selected terms associated with enriched biological processes and pathways based on the top DEGs (**C**). Heatmap showing normalized median gene expression of selected genes encoding type I IFNs and type I IFN receptor subunits across groups (**D**). mRNA expression of *Ifng* and *Ifnb1* relative to the housekeeping gene *Tbp* (cohorts of *n* = 5–7 mice) (**E**). Statistical significance was determined using a 2-sided unpaired Students *t* test for parametric, or Mann-Whitney test for nonparametric data. Heatmap showing normalized median gene expression of selected genes associated with type I IFN signaling (**F**). (**G**–**I**) Schematic representing the dosing strategy for SnMP (25 μmol/kg/daily) or respective vehicle and/or neutralizing anti-IFNAR1 antibody or control IgG in *Prf1^–/–^* *MMTV-PyMT* mice (**G**) and growth curves of treated tumors (**H**) and SnMP- or vehicle-treated tumors grown in mice on a *Prf1^–/–^* *Ifng^–/–^* *MMTV-PyMT* background (cohorts of *n* = 5–7 mice) (**I**). (**J**) Representative image of an FFPE tumor section from a *Prf1^–/–^* *MMTV-PyMT* mouse stained with DAPI (nuclei, blue), antibodies against F4/80 (red), and probed for *Ifnb1* mRNA (green). Scale bar: 50 μm. Panels **A** and **G** were created using BioRender software. Statistical analysis of tumor growth curves was performed using the “CompareGrowthCurves” function of the statmod software package and bar chart was compared using a Mann-Whitney test. Bar charts show the mean and the dots show individual data points from individual tumors and mice. Line charts display the mean and SEM. *P < 0.05; **P < 0.01.

To further characterize the TME, we performed gene expression analysis of tumors from *Prf1^+/+^* and *Prf1^–/–^* *MMTV-PyMT* mice treated with either vehicle or SnMP for 36 hours, using the NanoString nCounter platform with the Pan-Cancer Immune Profiling Panel (Figure 3, A–F). Gene expression differences were primarily associated with the mouse genotype (*Prf1* status), with 21 differentially expressed genes (DEGs), compared with only 2 DEGs associated with treatment (Figure 3B). Gene Ontology (GO) analysis revealed enrichment in immune response pathways, particularly those related to viral infection and type I IFN signaling, in *Prf1^–/–^* tumors (Figure 3C). Among IFN genes, *Ifnb1* was the most highly expressed type I IFN (Figure 3D), and its expression was higher than *Ifng* expression (Figure 3E). The TME in *Prf1^–/–^* *MMTV-PyMT* exhibited hallmarks of a chronic type I IFN response, including upregulation of multiple IFN-stimulated genes (ISGs) (Figure 3F). There was no evidence that SnMP treatment induced or significantly enhanced the type I IFN response.

To assess the functional relevance of type I IFN signaling in this TME, we treated tumor-bearing *Prf1^–/–^* mice with a neutralizing antibody against IFNAR1, a subunit of type I IFN receptor (Figure 3G). Blocking type I IFN signaling abrogated tumor control by SnMP (Figure 3H), indicating a role for type I IFN in mediating HO inhibitor efficacy. In contrast, HO inhibition remained effective in the absence of a type II IFN, IFN-γ, as demonstrated in *Ifng^–/–^* *Prf1^–/–^* *MMTV-PyMT* mice (Figure 3I), confirming a functionally relevant type I–skewed response in this TME.

Type I IFN responses are often induced via the cyclic GMP-AMP synthase (cGAS)/stimulator of IFN genes (STING) pathway (32), which can be activated by cytoplasmic DNA released from degrading micronuclei (33). However, quantitation of micronuclei revealed no difference in prevalence between *Prf1^+/+^* and *Prf1^–/–^* *MMTV-PyMT* tumors (Supplemental Figure 3F). To identify the cellular source of *Ifnb1* expression in the TME, we used RNAscope on *Prf1^–/–^* *MMTV-PyMT* tumor sections (Figure 3J and Supplemental Figure 3G). Type I IFNs have been described to be expressed by both tumor and stromal cell populations within the TME (34). While F4/80^+^ TAMs or viable tumor regions showed negligible *Ifnb1* expression, the most prominent signal was localized to regions of necrosis (Figure 3J and Supplemental Figure 3G), suggesting a nonimmune, damage-associated origin. Although the precise trigger of the chronic type I IFN response remains unidentified, our findings demonstrate that this response is essential for tumor control and underlies the TME’s sensitivity to HO inhibition.

### Type I IFN responses and HO-1 expression stratify prognosis in human breast cancer.

**Figure 4:**
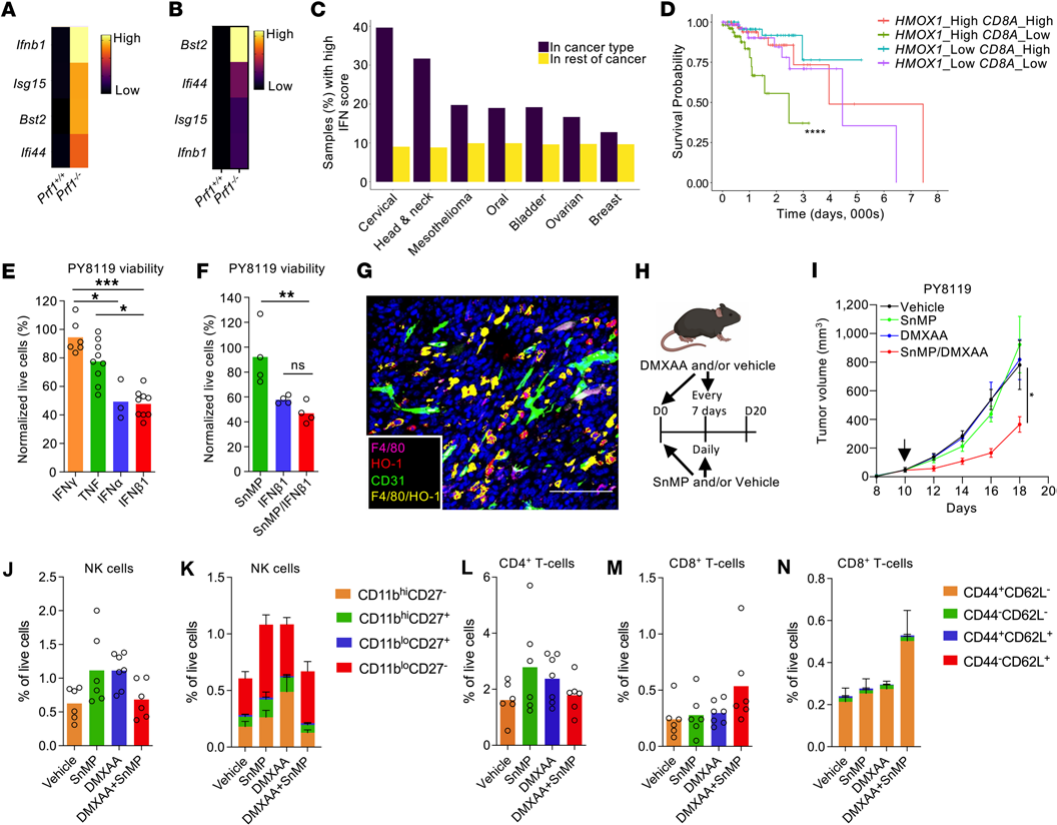
Therapeutically generated type I IFN responses synergize with HO inhibition to deliver tumor control. (**A** and **B**) Heatmap showing normalized median gene expression of selected genes encoding *Ifnb1* and type I IFN response genes across groups from NanoString nCounter analysis (**A**) and qRT-PCR for the indicated genes relative to *Tbp* (**B**) (*n* = 5 mice). (**C**) Fraction of TCGA samples within a cancer type that belong to top 10% type I IFN score TCGA samples with respect to the distribution of expression scores across all 9,062 TCGA samples (purple) compared with all TCGA cancer types (minus cancer type of interest, yellow). Indication *n* values available in Methods. (**D**) Survival curves of the 4 patient subgroups within the high type I IFN signature expressors (*n* = 252) generated using TCGA Breast Cancer data. Survival curves were compared using the log-rank (Mantel-Cox) test. (**E** and **F**) In vitro exposure of PY8119 cells to 10 ng/mL of the indicated cytokines (**E**) and 10 ng/mL IFN-β1 and/or SnMP (25 μM) (**F**), with viability assessed after 24 hours normalized to vehicle-treated cells (*n* = 4–9). (**G**) Representative image of a FFPE section of an orthotic PY8119 tumor stained with DAPI (nuclei, blue) and antibodies against CD31 (green), F4/80 (magenta), HO-1 (red), and F4/80 and HO-1 colocalization (yellow). Scale bar: 100 μm. (**H**–**N**) Schematic representing the dosing strategy for SnMP (25 μmol/kg/ daily), DMXAA (1.5 mg/kg/7 days), or vehicle in mice bearing PY8119 tumors (**H**), monitored for long-term growth (**I**). Alternatively, tumors were harvested after 36 hours, enzyme-dispersed, and cell populations analyzed by flow cytometry for the frequency of NK cells (**J**) or NK subsets (**K**), CD4^+^ (**L**) and CD8^+^ (**M**) T-cells and CD8^+^ T cell subsets (**N**) (cohorts of *n* = 6 mice). Panel **H** was created using BioRender software. Statistical analysis of tumor growth curves was performed using the “CompareGrowthCurves” function of the statmod software package. Statistical significance was determined using a Kruskal-Wallis test followed by Dunn’s multiple-comparison test. Bar charts show the mean and the dots show individual tumors and mice. Line chart displays the mean and SEM. *P < 0.05; **P < 0.01; ***P < 0.001; ****P < 0.0001.

Given the link between type I IFN responses and sensitivity to HO inhibition in our model, we explored whether similar TMEs exist in human cancers. We selected a panel of type I IFN–related genes, including, *Ifnb1*, *Isg15*, *Bst2*, and *Ifi44* that were significantly upregulated in *Prf1^–/–^* tumors compared to *Prf1^+/+^* tumors and had been validated using quantitative reverse transcription PCR (qRT-PCR) analyses (Figure 4, A and B). Using The Cancer Genome Atlas (TCGA), we surveyed various cancer types for elevated type I IFN pathway activity. The highest incidence was observed in cervical and head and neck cancers (Figure 4C). Notably, these cancers are frequently driven by human papillomavirus infection, known to trigger antiviral type I IFN responses (35). Breast cancer was also enriched for TMEs with a type I IFN signature (Figure 4C).

To assess the clinical relevance in breast cancer, we examined the association between type I IFN signaling, CD8^+^ T cell infiltration (via *CD8A*), and HO-1 (*HMOX1*) expression. Across the cohort, *HMOX1* expression positively correlated with both *CD8A* and type I IFN activity (Supplemental Figure 4A). We stratified tumors based on high type I IFN gene expression and evaluated survival outcomes. Patients with a *CD8A*^hi^*HMOX1*^lo^ profile had the most favorable prognosis, whereas those with a *CD8A*^lo^*HMOX1*^hi^ profile had the poorest outcomes (Figure 4D). Notably, *HMOX1* alone was not prognostic (Supplemental Figure 4A), nor was *HMOX1* and *CD8A* prognostic in tumors with the lowest expression of type I IFN response genes (Supplemental Figure 4B). These findings suggest that in human cancers, HO-1 activity may contribute to immune evasion and tumor progression in TMEs with chronic type I IFN signaling.

### STING agonists synergize with HO inhibitors to control tumor growth.

Since not all tumors exhibit a type I IFN signature, we investigated whether therapeutically inducing this response could sensitize tumors to HO inhibition. STING agonists, which are currently in clinical trials across multiple tumor types (36), offer a promising strategy to trigger type I IFN responses in the TME. To test this, we used an orthotopic breast cancer model based on the PY8119 cell line, which was originally derived from a *MMTV-PyMT* tumor from a pure C57BL/6 mouse background (37).

In vitro*,* PY8119 cells were sensitive to both IFN-α1 and -β1 (Figure 4E), but this effect was not enhanced by SnMP (Figure 4F), suggesting a lack of intrinsic synergy between HO inhibition and type I IFN signaling in the absence of the full TME and an active immune response. In vivo*,* orthotopically implanted PY8119 tumors in syngeneic C57BL/6 mice were characterized ex vivo. Immunofluorescence analysis revealed that HO-1 was expressed by PvTAMs, similar to spontaneous *MMTV-PyMT* tumors, although the vasculature appeared less organized in this model (Figure 4G).

Mice bearing established PY8119 tumors were treated with the STING agonist 5,6-dimethylxanthenone-4-acetic acid (DMXAA) and/or SnMP (Figure 4H). Neither DMXAA nor SnMP alone affected tumor growth, but their combination significantly reduced tumor burden (Figure 4I), indicating that HO activity may act as a resistance mechanism to type I IFN–driven antitumor responses. To investigate the cellular changes in the TME, in a separate experiment, we analyzed enzyme-digested tumors 36 hours after treatment initiation using flow cytometry. Similar to observations using *MMTV-PyMT* mice, there was no change in the abundance of NK cells (Figure 4J) or their subsets (Figure 4K), CD4^+^ T cells (Figure 4L), CD8^+^ T cells (Figure 4M), or their subsets (Figure 4N), nor the broader stromal composition of these tumors (Supplemental Figure 5). However, these findings reveal a link between type I IFN responses in the TME and HO activity as a resistance mechanism. These findings provide strong translational rationale for combining HO inhibitors with STING agonists to enhance therapeutic efficacy.

## Discussion

This study describes a TME that is responsive to HO inhibitors and characterized by a chronic type I IFN response, which plays a direct role in mediating tumor control. The role of type I IFN responses in the TME remains complex and context dependent (32, 38–40). While chronic type I IFN signaling is often associated with immunosuppression (41), transient exposure can promote immunostimulatory effects (42).

HO-1 is primarily expressed by PvTAMs in *MMTV-PyMT* tumors (3). Our findings suggest that HO activity, likely associated with PvTAMs, modulates immune function and influences the outcome of chronic type I IFN responses within the TME. Previous studies have shown that HO-1 can directly suppress type I IFN responses by modulating IFN regulatory factors 3/7 (IRF3/7) (43, 44). CO has the potential to modulate downstream type I IFN signaling through its ability to suppress STAT1 activation (45), a key component of the type I IFN receptor signaling pathway (46).

However, in this study, HO inhibition with SnMP did not alter the abundance of LYVE-1^+^ TAMs or directly induce or amplify the type I IFN responses in the TME. This suggests that HO activity influences the TME’s response to chronic type I IFN rather than initiating it. In *Prf1^–/–^* *MMTV-PyMT* tumors, *Ifnb1* expression was not associated with a specific cell type but was predominantly localized to necrotic tumor regions. Since type I IFNs can be induced by extracellular DNA released during necrotic cell death via the cGAS/STING pathway (47, 48), this may explain their localization. Although the cause of the heightened type I IFN response in *Prf1^–/–^* tumors remains unclear, it may contribute to the delayed tumor onset observed in these mice, suggesting incomplete suppression of this response by HO-1.

Sustained type I IFN responses in the TME have been linked to resistance to immune checkpoint inhibitors (49–51). Similarly, HO-1 has been described to function like an immune checkpoint (15), given its role in suppressing the adaptive immune response against cancer (3, 12, 15, 52). Tumor control in *Prf1^–/–^* *MMTV-PyMT* mice treated with SnMP was dependent on CD8^+^ T cells and NK cells but independent of their expression of perforin or IFN-γ. This implies possible involvement of alternative cytotoxic pathways, such as Fas/Fas-Ligand, TNF-related apoptosis-inducing ligand (TRAIL) (53–55), or TNF-mediated bystander killing (56).

Type I IFNs are known to stimulate NK cell activity (57–59), enhance T cell responses indirectly by activating antigen-presenting cells (60), and protect T cells from NK cell–mediated killing (61). The fact that both CD8^+^ T and NK cells were required for tumor control suggests either cooperative killing (62, 63), a “self/missing-self”/nonclassical killing mechanism, or bystander effect (64–66).

Understanding the TME and using this knowledge to guide therapy selection is critical to improving immunotherapy response rates. There is also a clear need for more personalized strategies. Our study shows that a subset of human cancers, including breast cancer, exhibit signs of chronic type I IFN signaling. We demonstrate that high *HMOX1* and low *CD8A* expression in breast cancer is associated with poorer outcomes, suggesting that these patients may benefit from HO inhibition to overcome immunosuppression in the TME.

Building on this concept, we propose a therapeutic strategy for tumors that lack an endogenous type I response, utilizing STING agonists to reprogram the TME to produce a type I IFN response, rendering tumors more susceptible to HO inhibitors. STING activation has been shown to induce the activation and cytotoxicity of both NK (59) and CD8^+^ T cells (67). Additionally, STING activation can promote T cell infiltration into the TME (68, 69), but it may also induce endoplasmic reticulum stress responses that lead to T cell death (70). In our study, however, we observed no significant changes in the CD8^+^ T cell abundance within the TME. Although STING agonists are currently under active clinical development, their efficacy as monotherapies has generally been limited (71). Our findings support a translational rationale for combining STING agonists with HO inhibitors to enhance antitumor immunity and improve therapeutic outcomes.

## Methods

### Sex as a biological variable.

The current study utilized exclusively female mice. The spontaneous *MMTV-PyMT* model of breast cancer utilizes a mammary specific promoter (*MMTV*) to drive expression of the viral oncogene *PyMT*. As such, this transgenic model is restricted to female animals. The PY8119 cell line, used in this study, was derived from a spontaneous *MMTV-PyMT* tumor and implanting these cells back into a mammary fat pad of female mice represented an orthotopic model setting.

### Mice.

WT mice used in this study were female and 4–6 weeks-old on a C57BL/6 background and obtained from Charles River. Transgenic mice (knockout; KO) used in the study included homozygous *Prf1^–/–^* (C57BL/6-*Prf1^tm1Sdz^*/J) and *Ifng^–/–^* (B6.129S7-*Ifng^tm1Ts^*/J) and were on a C57BL/6 background, and *MMTV-PyMT* mice were on FVB/N background and obtained from The Jackson Laboratory. Where indicated, female KO mice were crossed with male *MMTV-PyMT* mice and the F2 homozygous KO or F2 WT offspring were used experimentally. Where *MMTV-PyMT* mice were to be used and not compared to a KO strain, male *MMTV-PyMT* mice were bred with WT FVB/N females and the F1 offspring were used experimentally. Cohort sizes were informed by prior studies (11, 15, 16, 72). All mice used for experiments were female and randomly assigned to treatment groups. Mice weighed approximately 21–26 g when tumors became palpable. Experiments were performed in at least duplicate and for spontaneous *MMTV-PyMT* tumor studies individual mice were collected on separate days and all data points are presented.

### Cell lines.

PY8119 cells were a gift from Toby Lawrence (King’s College London). Cell lines were confirmed to be mycoplasma free using the MycoAlert Mycoplasma Detection Kit (Lonza) and were cultured in F-12K Medium (Kaighn’s modification of Ham’s F-12 medium) (Gibco) supplemented with 5% FCS (Thermo Fisher Scientific), 0.1% MITO+ Serum Extender (Corning), and 1% penicillin/streptomycin (Sigma-Aldrich). Luc/eGFP/OVA-expressing PY8119 (PY8119/Luc) were generated by transducing PY8119 cells with the pMIG retroviral vector modified to contain luciferase (Luc), enhanced green fluorescent protein (eGFP), and chicken egg ovalbumin (OVA) adapted from Kosti et al. (73). In brief, Phoenix-ECO retrovirus-producing cells were transfected with the desired plasmid and pCL-Eco retrovirus packaging vector using FuGENE Transfection Reagent (Promega) in serum-free Opti-MEM (Gibco). On the following day, culture media were replaced with fresh complete Opti-MEM. After 24 hours, conditioned media containing retrovirus were collected and used together with 4 μg/mL Polybrene (Santa Cruz Biotechnology) for the transduction of PY8119 cells. Transduced cells were purified by fluorescence-activated cell sorting (FACS) by selectively gating for eGFP-expressing cells and later expanded in complete F-12K media.

### Tumor studies.

For the orthotopic tumors, 2.5 × 10^5^ PY8119 cells in 100 μL RPMI were subcutaneously (s.c.) injected into the mammary fat pad of syngeneic C57BL/6 female mice. When tumors became palpable, volumes were measured every 2 days using digital caliper measurements of the long (L) and short (S) dimensions of the tumor. Tumor volume was established using the following equation: volume = (S^2^ × L)/2. In studies using *MMTV-PyMT* mice, the primary tumor growth is presented. For drug treatments, drugs were freshly prepared on the day of injection and administered by intraperitoneal (i.p.) injection using a 26-G needle. Tin (IV) mesoporphyrin IX dichloride (SnMP; Frontier Scientific) was dissolved and administered as previously described at a dose of 25 μmol/kg/day (16). A stock solution of 5,6-dimethylxanthenone-4-acetic acid (DMXAA, Cayman Chemical) was generated by dissolving in DMSO at 2 mg/mL and stored at –80 °C until used. On the day of injection, the stock solution was freshly dissolved in sterile DPBS and administered i.p. at 1.5 mg/kg/ once every 7 days. In vivo immune depletion was achieved through i.p. administration of depleting antibodies. For blocking of IFN-α receptor 1 (IFNAR1), mice were injected with 100 μg of neutralizing anti-IFNAR1 monoclonal antibody (MAR1-5A3, 2B Scientific; isotype: mouse IgG1, κ) 3 times per week, with a loading dose 2 days prior to treatment initiation with SnMP. For depletion of CD8^+^ T cells or NK cells, mice were injected with 400 μg of GoInVivo purified anti–mouse CD8α (53-6.7, BioLegend) or Ultra-LEAF purified anti–mouse NK-1.1 antibody (PK136, BioLegend), respectively, or GoInVivo purified Rat IgG2a as control (RTK2758, BioLegend) every 4 days, starting with a loading dose 2 days prior to the initiation of SnMP. At the end of in vivo studies, tumors were excised and tissue was enzyme digested to release single cells as previously described (72). In brief, tissues were minced using scalpels, and then single cells were liberated by incubation for 60 minutes at 37°C with 1 mg/mL Collagenase I from *Clostridium histolyticum* (Sigma-Aldrich) and 0.1 mg/mL Deoxyribonuclease I (AppliChem) in RPMI (Gibco). Released cells were then passed through a 70 μm cell strainer prior to staining for flow cytometry analyses. Viable cells were numerated using a hemocytometer with trypan blue (Sigma-Aldrich) exclusion.

### Flow cytometry.

Flow cytometry was performed as previously described (16). The following antibodies against the indicated antigen were used at 1 μg/mL unless stated otherwise: CD103 PE, PE-Cyanine5 (2E7, BioLegend); CD11b BV421, BV510, and APC780 (M1/70, BioLegend); CD11c APC-eFluor 780, APC, and FITC (N418, BioLegend); CD178 (FasL) PE (MFL3, BioLegend); CD19 FITC, BV711 (1D3/CD19, BioLegend); CD19 APC and BV421 (6D5, BioLegend); CD206 APC (FAB2535A, Bio-Techne); CD206 FITC, PE-Cyanine7 (C068C2, BioLegend); CD27 PE, Alexa Fluor 700 (LG.3A10, BioLegend); CD27 APC-eFluor 780 (LG.7F9, Invitrogen); CD273 (PD-L2) APC (TY25, BioLegend); CD31 FITC, Alexa Fluor 700 (390, BioLegend); CD314 (NKG2D) PE (C7, BioLegend); CD335 (NKp46) eFluor 450 (29A1.4, Invitrogen); CD3ε PE, PE-Cyanine7 (17A2, BioLegend); CD3ε APC, APC/Cyanine7, and BV421 (145-2C11, BioLegend); CD4 FITC, APC (RM4-5, BioLegend); CD44 BV711 (IM7, BioLegend); CD45 APC-eFluor 780, BV510, and BV785 (30-F11, BioLegend); CD49A PE (HA31/8, BD Biosciences); CD49b APC-Cyanine7 (DX5, BioLegend); CD62L, APC-Cyanine7 (MEL-14, BioLegend); CD69 PE-Cyanine5, APC (H1.2F3, BioLegend); CD8α eFluor 450, BV421, and FITC (53-6.7, BioLegend); CD8β FITC (YTS156.7.7, BioLegend); CD8β FITC (H35-17.2, Invitrogen); CD90.1 eFluor 450 (HIS51, Thermo Fisher Scientific); CD90.1 BV510 (OX-7, BioLegend); CD90.2 eFluor 450, BV510 (53-2.1, BioLegend); CD95 (Fas) PE (SA367H8, BioLegend); F4/80 APC-eFluor 780, APC/Cyanine7, BV421, and FITC (BM8, BioLegend); FOXP3 PE-Cyanine5 (FJK-16s, Invitrogen), granzyme B PE (GB11, Thermo Fisher Scientific; 1:10); H-2Db PE (KH95, BioLegend); H-2Dq H-2Lq BV510 (KH117, BD Biosciences); H-2Kb Pacific Blue (AF6-88.5, BioLegend); IFN-γ PE (XMG1.2, BioLegend); Ki-67 PE-Cyanine7, Alexa Fluor 700 (SolA15, Invitrogen); Ly-49AB6 PE (A1/Ly49A, BioLegend), Ly-6C FITC, PE, BV421 (HK1.4, BioLegend); Ly-6G/Ly-6C (Gr-1) FITC (RB6-8C5, BioLegend); Ly-6G BV563 (1A8, BD Biosciences); MHC Class II (I-A/I-E) BV421, FITC, PE, APC (M5/114.15.2, BioLegend); NK-1.1 APC, PE (PK136, BioLegend); NK-1.1 APC (PK136), TER-119 PerCP-Cyanine5.5 (TER-119, BioLegend); TNF-α PE (MP6-XT22, BioLegend); and TNF-α APC (MP6-XT22, Thermo Fisher Scientific). Where stated, the following corresponding isotype control antibodies at equivalent concentrations to that of the test stain were used: IgG1 PE and APC (RTK2071, BioLegend), IgG2a BV510 and Pacific Blue (RTK2758, BioLegend), and IgG2b BV421 (RTK4530, BioLegend). Intracellular staining was performed as previously described (16). Dead cells and red blood cells were excluded using 1 μg/mL 7-amino actinomycin D (7AAD; Sigma-Aldrich) or Zombie UV (BioLegend)/Fixable Viability Dye eFluor 780 (Invitrogen) alongside anti–Ter-119 PerCP-Cy5.5 (Ter-119, Invitrogen). Data were collected on a BD FACSCanto II (BD Biosciences) or CytoFLEX (Beckman Coulter). Data were analyzed using FlowJo software (Treestar Inc.).

### Immunofluorescence.

Mouse mammary tumors were fixed overnight in 4% paraformaldehyde, followed by overnight dehydration in 30% sucrose prior to embedding in Optimal Cutting Temperature (OCT; VWR Chemicals) and snap freezing in liquid nitrogen. Frozen sections from these tumors were fixed in 4 % paraformaldehyde in DPBS for 10 minutes at room temperature and were washed in Tris-buffered saline (100 mM Tris, 140 mM NaCl) with 0.05% Tween 20, pH 7.4 (TBST). Sections from FFPE human in invasive ductal carcinoma tissue tumor tissue were acquired from the King’s Health Partners Biobank. FFPE sections were deparaffinized in xylenes (3 washes, 5 minutes each) and rehydrated in an ethanol/water gradient series: 100% ethanol, 95% ethanol, and finally water (all 2 washes, 5 minutes each wash). Epitopes were unmasked using 20 mg/mL Proteinase K (25530049, Thermo Fisher Scientific) and incubated for 3 minutes at room temperature. The slides were then washed with TBS-T (3 washes, 3 minutes each). Mouse and human sections were blocked with TBS-T, 10% donkey serum (Sigma-Aldrich), and 0.2% Triton X-100. Immunofluorescence was performed as previously described (11). For mouse tissues, antibodies against the following targets and their dilutions were used as follows: CD31 (1:100; AF3628, R&D Systems), F4/80 (1:100; C1:A3-1, Bio-Rad), HO-1 (1:100; 10701-1-AP, Proteintech), and CD3 (1:100; SP7, Abcam). For human tissues, antibodies against the following targets and their dilutions were used as follows: CD31–Alexa Fluor 647 (10 μg/mL; clone JCC/70A, ab215912, Abcam), CD68-Alexa Fluor 594 (0.5 μg/mL; clone KPI, sc-20060, Santa Cruz Biotechnology), and HO-1 (1:100; 10701-1-AP, Proteintech). Unconjugated primary antibodies were detected using antigen-specific donkey IgG, used at 1:200 dilution: Alexa Fluor 488 anti-rabbit IgG, Alexa Fluor 488 anti-goat IgG, Alexa Fluor 568 anti-rabbit IgG, Alexa Fluor 568 anti-goat IgG, Alexa Fluor 647 anti-rabbit IgG, Alexa Fluor 647 anti-rat IgG, Alexa Fluor 532 anti-rabbit IgG (Thermo Fisher Scientific), and Alexa Flour 594 anti-rat IgG (Abcam). RNAscope was performed on FFPE *MMTV-PyMT* tumor sections as per manufacturer’s instructions using the RNAscope Multiplex Fluorescent Reagent Kit v2 Assay (Bio-Techne, 323100-USM). Mm-Ifnb1 (406531, Advanced Cell Diagnostics, Inc.) probe was used and was detected using TSA Vivid Fluorophore 520 (1:1000; Bio-Techne). Subsequently, immunofluorescence imaging was performed as described above. Nuclei were stained using 1.25 μg/mL 4′,6-diamidino-2-phenylindole,dihydrochloride (DAPI) (Thermo Fisher Scientific). Nuclei quantification based on immunofluorescence images was performed using an unsupervised object detection pipeline developed using CellProfiler (Broad Institute of MIT and Harvard). Immunofluorescence images of *Prf1^+/+^* and *Prf1^–/–^* *MMTV-PyMT* tumor sections (*n* = 6), with 5 randomly selected areas per tumor, were processed. The DAPI channel was extracted and converted to maximum intensity *Z*-stack projections in grayscale. Individual nuclei and micronuclei were detected using the IdentifyPrimaryObjects module with Otsu adaptive thresholding. The MeasureObjectSizeShape module was applied to calculate the area of each detected object in pixel units. The size range in pixel units used to classify objects as either nuclei or micronuclei was manually inferred from sampled images by assessing the area of example objects subjectively classified as micronuclei, nuclei, or artifacts. Objects with an area of 50–400 pixel units were classified as micronuclei, 800–12,500 pixel units as intact nuclei, smaller objects as artifacts or debris, and larger objects as clusters of multiple overlapping nuclei, which were excluded from further analysis. Images were acquired using a Nikon Eclipse Ti-E inverted spinning disk confocal with associated NIS Elements software. Full-section images were acquired using a NanoZoomer Digital Slide Scanner (Hamamatsu). Quantitative data were acquired from the images using NIS Elements software.

### qRT-PCR.

mRNA was extracted and qRT-PCR was performed as previously described (7) or the TRIzol method using the EXPRESS one-step Superscript RT PCR kit and the following primers/probes purchased from Thermo Fisher Scientific: *Bst2* Mm01609165_g1, *Cxcl9* Mm00434946_m1, *Cxcl10* Mm00445235_m1, *Cxcl11* Mm00444662_m1, *Hmox1* Mm00516005_m1, *Ifi44* Mm00505670_m1, *Ifnb1* Mm00439552_s1, *Ifng* Mm01168134_m1, *Isg15* Mm01705338_s1, and *Tbp* Mm01277045_m1. Expression of all genes is represented relative to the housekeeping gene Tata-binding protein (*Tbp*). Assays were performed using a QuantStudio 7 Real-Time PCR instrument (Thermo Fisher Scientific).

### Cell viability assay.

PY8119/Luc cells (1 × 10^4^/well in 100 μL) were seeded into 96-well tissue culture plates in complete F-12K medium. After 24 hours, cells were treated with recombinant cytokines IFN-γ, IFN-β1, or TNF (Bio-Techne) at a final concentration of 10 ng/mL with or without 25 μM SnMP for 24 hours at 37°C. Cell viability was determined by luciferase quantification following the addition of 1 μL of 15 mg/mL XenoLight D-luciferin (PerkinElmer) in DPBS per well. After a 10-minute incubation at 37°C, luminescence was quantified using a CLARIOstar Plus plate reader (BMG Labtech) and values from the treated samples were normalized to those from vehicle control (DPBS). At least 3 independent experiments were performed.

### NanoString nCounter analysis.

Excised *MMTV-PyMT* tumor tissue samples were placed in RNAlater Stabilization Solution (Thermo Fisher Scientific) at 4°C for 24 hours, and subsequently transferred to and stored at –80°C until used for RNA extraction. RNA from *MMTV-PyMT* tumors was isolated using TRIzol (Thermo Fisher Scientific) according to the manufacturer’s protocol. Isolated RNA was quantified with the NanoDrop 2000 spectrophotometer (Thermo Fisher Scientific) or using the Qubit RNA HS Assay Kit as per the manufacturer’s recommendations (Thermo Fisher Scientific). RNA samples were analyzed using the RNA 6000 Nano Kit (Agilent Technologies) and the RNA integrity was assessed based on the RNA integrity number (RIN) determined using the Agilent 2100 Bioanalyzer. A multiplex analysis of the expression of 770 genes including 40 housekeeping genes was performed using the nCounter Pan-Cancer Immune Profiling Panel and the nCounter platform, loading 80 ng of total RNA for each sample according to the manufacturer’s recommendations (NanoString Technologies). The NanoString data were normalized to housekeeping genes using the remove unwanted variation (RUV) pipeline using the RUVSeq package in R (74). The subsequent differential expression analysis of the NanoString data was performed with DESeq2 package in R (75). Enriched pathways were identified based on DEGs using gProfiler (76) (http://www.biit.cs.ut.ee/gprofiler/). We used pathways gene sets from the “biological processes” (GO:BP) of Gene Ontology (http://www.geneontology.org/). Benjamini-Hochberg correction was applied to the *P* values to adjust for multiple hypothesis testing. Genes with adjusted *P* values lower than 0.05 were considered significant.

### Human transcriptomic expression analysis and survival.

Single-sample gene set enrichment analysis (ssGSEA) was used to compute normalized enrichment scores (NES) of type I IFN signature genes across 34 cancer types from TCGA. The top 10% of all 9,062 TCGA samples by type I IFN NES was arbitrarily defined as having elevated enrichment of type I IFN pathway. Subsequently, for each of the 32 cancer types, their proportion of samples contributing to the pan-cancer top 10% type I IFN group was compared to the background proportion by Fisher’s exact test with Benjamini-Hochberg correction across 34 cancer types. The sample distribution was the following: cervical squamous cell carcinoma and endocervical adenocarcinoma (CESC, *n* = 290), head and neck squamous cell carcinoma (HNSC, *n* = 467), mesothelioma (MESO, *n* = 81), oral squamous cell carcinoma (OSCC, *n* = 79), bladder urothelial carcinoma (BLCA, *n* = 370), ovarian serous cystadenocarcinoma (OV, *n* = 360), and breast invasive carcinoma (BRCA, *n* = 1018).

For correlation analysis, we conducted Pearson’s correlation using the *ggpubr* package in R for *HMOX1* expression with *CD8A* expression and IFN score for all patients (*n* = 1083), low expressors (*n* = 225), and high expressors (*n* = 253). The IFN score for correlation analysis was obtained by summing up *BST2*, *IFI44*, *ISG15*, and *IFNB1* expression. We also conducted survival analyses for *HMOX1*-High and *HMOX1*-Low subgroups across all the 3 groups.

For survival analysis of patients with breast cancer, the processed transcriptomics count data for 1595 samples were downloaded from https://portal.gdc.cancer.gov/ The primary tumor samples from the TCGA-BRCA project were downloaded, which gave 1111 samples. Samples having duplicates were removed to include a total of 1084 unique patient tumor samples in the final analysis. Out of 1084 patients, 932 patients were alive at the last follow-up, 151 patients had died, and 1 had unreported vital status. A total of 1083 patients were stratified into low and high expressors for the 4 signature genes (*BST2*, *IFI44*, *ISG15*, and *IFNB1*) individually using the median value for each. From this, 252 patients who were high expressors for all the 4 genes were extracted. To investigate the association of *CD8A* and *HMOX1* expression with survival among these high expressors, we substratified them into the 4 following groups: (a) *HMOX1*-High-*CD8A*-High (*n* = 71), (b) *HMOX1*-High-*CD8A*-Low (*n* = 55), (c) *HMOX1*-Low-*CD8A*-High (*n* = 55), and (d) *HMOX1*-Low-*CD8A*-Low (*n* = 71). We also removed 225 patients who were low expressors for all 4 genes. To investigate the association of *CD8A* and *HMOX1* with survival among these low expressors, we substratified them into the 4 following groups: (a) *HMOX1*-High-*CD8A*-High (*n* = 61), (b) *HMOX1*-High-*CD8A*-Low (*n* = 51), (c) *HMOX1*-Low-*CD8A*-High (*n* = 51), and (d) *HMOX1*-Low-*CD8A*-Low (*n* = 62). Kaplan-Meier survival curves were estimated using the *survfit* function from the *survival* package in R. The survival curves were drawn using the *ggsurvplot* function from the *survminer* package in R.

### Statistics.

Normality and homogeneity of variance were determined using a Shapiro-Wilk normality test and an *F* test, respectively. Statistical significance was then determined using GraphPad Prism 8 software. For comparison between 2 groups, a 2-sided, unpaired Students *t* test was used for parametric data, or a Mann-Whitney test for nonparametric data. A Welch’s correction was applied when comparing groups with unequal variances. For comparisons involving more than 2 groups, a Kruskal-Wallis test followed by Dunn’s multiple-comparison test was used for nonparametric data. Survival curves were compared using the log-rank (Mantel-Cox) test. Statistical analysis of tumor growth curves was performed using the “CompareGrowthCurves” function of the statmod software package (77). No outliers were excluded from any data presented.

### Study approval.

All experiments involving animals were approved by the Animal and Welfare and Ethical Review Board of King’s College London and the Home Office UK. Human breast adenocarcinoma tissue was obtained with informed consent under ethical approval from the King’s Health Partners Cancer Biobank (REC reference 12/EE/0493).

### Data availability.

For survival analysis of patients with breast cancer, the processed transcriptomics count data for 1595 samples were downloaded from https://portal.gdc.cancer.gov/ The NanoString nCounter data are available in the NCBI Gene Expression Omnibus (https://www.ncbi.nlm.nih.gov/geo/query/acc.cgi?acc=GSE279433). The authors declare that all other data supporting the findings of this study are available within the paper and its supplemental information files. Any additional information required to reanalyze the data reported in this paper is available from the lead contact upon request. Values for all data points in graphs are reported in the Supporting Data Values file.

## Author contributions

DS, TSC, and JNA conceived the project, designed the approach, performed experiments, interpreted the data, and wrote the manuscript. J Sarkar, JWO, KTF, JEA, CAW, MRK, KC, SA, WM, DDY, and JR designed the approach, performed experiments, and interpreted the data. CEG, FDC, RB, J Spicer, and AG designed experiments, interpreted the data, and provided key expertise.

## Supplementary Material

Supplemental data

Supplemental data set 1

Supporting data values

## Version 1 07/08/2025

In-Press Preview

## Version 2 08/22/2025

Electronic publication

## References

[1] Mariathasan S (2018). TGFβ attenuates tumour response to PD-L1 blockade by contributing to exclusion of T cells. Nature.

[2] Cascio S (2021). Cancer-associated MSC drive tumor immune exclusion and resistance to immunotherapy, which can be overcome by Hedgehog inhibition. Sci Adv.

[3] Anstee JE (2023). LYVE-1^+^ macrophages form a collaborative CCR5-dependent perivascular niche that influences chemotherapy responses in murine breast cancer. Dev Cell.

[4] de Visser KE, Joyce JA (2023). The evolving tumor microenvironment: from cancer initiation to metastatic outgrowth. Cancer Cell.

[5] Bagaev A (2021). Conserved pan-cancer microenvironment subtypes predict response to immunotherapy. Cancer Cell.

[6] Larkin J (2015). Combined nivolumab and ipilimumab or monotherapy in untreated melanoma. N Engl J Med.

[7] Jain RK (2001). Normalizing tumor vasculature with anti-angiogenic therapy: a new paradigm for combination therapy. Nat Med.

[8] Teicher BA (1996). A systems approach to cancer therapy. (Antioncogenics + standard cytotoxics-->mechanism(s) of interaction). Cancer Metastasis Rev.

[9] Luu Hoang KN (2021). The diverse roles of heme oxygenase-1 in tumor progression. Front Immunol.

[10] Consonni FM (2021). Heme catabolism by tumor-associated macrophages controls metastasis formation. Nat Immunol.

[11] Muliaditan T (2018). Macrophages are exploited from an innate wound healing response to facilitate cancer metastasis. Nat Commun.

[12] Di Biase S (2016). Fasting-mimicking diet reduces HO-1 to promote T cell-mediated tumor cytotoxicity. Cancer Cell.

[13] Berberat PO (2005). Inhibition of heme oxygenase-1 increases responsiveness of pancreatic cancer cells to anticancer treatment. Clin Cancer Res.

[14] Gozzelino R (2010). Mechanisms of cell protection by heme oxygenase-1. Annu Rev Pharmacol Toxicol.

[15] Muliaditan T (2018). Repurposing tin mesoporphyrin as an immune checkpoint inhibitor shows therapeutic efficacy in preclinical models of cancer. Clin Cancer Res.

[16] Arnold JN (2014). Tumoral immune suppression by macrophages expressing fibroblast activation protein-α and heme oxygenase-1. Cancer Immunol Res.

[17] Hughes R (2015). Perivascular M2 macrophages stimulate tumor relapse after chemotherapy. Cancer Res.

[18] Lapenna A (2018). Perivascular macrophages in health and disease. Nat Rev Immunol.

[19] Lewis CE (2016). The multifaceted role of perivascular macrophages in tumors. Cancer Cell.

[20] Opzoomer JW (2019). Cytotoxic chemotherapy as an immune stimulus: a molecular perspective on turning up the immunological heat on cancer. Front Immunol.

[21] Voskoboinik I (2015). Perforin and granzymes: function, dysfunction and human pathology. Nat Rev Immunol.

[22] Brennan AJ (2010). Perforin deficiency and susceptibility to cancer. Cell Death Differ.

[23] Van den Broek ME (1996). Decreased tumor surveillance in perforin-deficient mice. J Exp Med.

[24] Palladini A (2018). Cancer immunoprevention: from mice to early clinical trials. BMC Immunol.

[25] Koebel CM (2007). Adaptive immunity maintains occult cancer in an equilibrium state. Nature.

[26] Valaes T (1994). Control of jaundice in preterm newborns by an inhibitor of bilirubin production: studies with tin-mesoporphyrin. Pediatrics.

[27] Opzoomer JW (2021). Macrophages orchestrate the expansion of a proangiogenic perivascular niche during cancer progression. Sci Adv.

[28] Ovadya Y (2018). Impaired immune surveillance accelerates accumulation of senescent cells and aging. Nat Commun.

[29] Angell TE (2014). MHC class I loss is a frequent mechanism of immune escape in papillary thyroid cancer that is reversed by interferon and selumetinib treatment in vitro. Clin Cancer Res.

[30] McKenzie MD (2006). Perforin and Fas induced by IFNgamma and TNFalpha mediate beta cell death by OT-I CTL. Int Immunol.

[31] Bald T (2020). The NK cell-cancer cycle: advances and new challenges in NK cell-based immunotherapies. Nat Immunol.

[32] Samson N, Ablasser A (2022). The cGAS-STING pathway and cancer. Nat Cancer.

[33] Mackenzie KJ (2017). cGAS surveillance of micronuclei links genome instability to innate immunity. Nature.

[34] Boukhaled GM (2021). Opposing roles of type I interferons in cancer immunity. Annu Rev Pathol.

[35] Jensen JE (2024). Human papillomavirus and associated cancers: a review. Viruses.

[36] Le Naour J (2020). Trial watch: STING agonists in cancer therapy. Oncoimmunology.

[37] Gibby K (2012). Early vascular deficits are correlated with delayed mammary tumorigenesis in the MMTV-PyMT transgenic mouse following genetic ablation of the NG2 proteoglycan. Breast Cancer Res.

[38] Gulen MF (2017). Signalling strength determines proapoptotic functions of STING. Nat Commun.

[39] Zemek RM (2022). Temporally restricted activation of IFNβ signaling underlies response to immune checkpoint therapy in mice. Nat Commun.

[40] Lam KC (2021). Microbiota triggers STING-type I IFN-dependent monocyte reprogramming of the tumor microenvironment. Cell.

[41] Liang H (2017). Host STING-dependent MDSC mobilization drives extrinsic radiation resistance. Nat Commun.

[42] Corrales L (2015). Direct activation of STING in the tumor microenvironment leads to potent and systemic tumor regression and immunity. Cell Rep.

[43] Wu M (2024). Bi-directional regulation of type I interferon signaling by heme oxygenase-1. iScience.

[44] Tzima S (2009). Myeloid heme oxygenase-1 regulates innate immunity and autoimmunity by modulating IFN-beta production. J Exp Med.

[45] Zhang X (2005). Carbon monoxide differentially modulates STAT1 and STAT3 and inhibits apoptosis via a phosphatidylinositol 3-kinase/Akt and p38 kinase-dependent STAT3 pathway during anoxia-reoxygenation injury. J Biol Chem.

[46] Yu R (2022). Type I interferon-mediated tumor immunity and its role in immunotherapy. Cell Mol Life Sci.

[47] Deng L (2014). STING-dependent cytosolic DNA sensing promotes radiation-induced type I interferon-dependent antitumor immunity in immunogenic tumors. Immunity.

[48] King KR (2017). IRF3 and type I interferons fuel a fatal response to myocardial infarction. Nat Med.

[49] Qiu J (2023). Cancer cells resistant to immune checkpoint blockade acquire interferon-associated epigenetic memory to sustain T cell dysfunction. Nat Cancer.

[50] Jacquelot N (2019). Sustained type I interferon signaling as a mechanism of resistance to PD-1 blockade. Cell Res.

[51] Acha-Sagredo A (2025). A constitutive interferon-high immunophenotype defines response to immunotherapy in colorectal cancer. Cancer Cell.

[52] Alaluf E (2020). Heme oxygenase-1 orchestrates the immunosuppressive program of tumor-associated macrophages. JCI Insight.

[53] Golstein P, Griffiths GM (2018). An early history of T cell-mediated cytotoxicity. Nat Rev Immunol.

[54] Prager I (2019). NK cells switch from granzyme B to death receptor-mediated cytotoxicity during serial killing. J Exp Med.

[55] Bradley M (1998). Role of spontaneous and interleukin-2-induced natural killer cell activity in the cytotoxicity and rejection of Fas+ and Fas- tumor cells. Blood.

[56] Kearney CJ (2018). Tumor immune evasion arises through loss of TNF sensitivity. Sci Immunol.

[57] Biron CA (2002). Innate immune responses to LCMV infections: natural killer cells and cytokines. Curr Top Microbiol Immunol.

[58] Swann JB (2007). Type I IFN contributes to NK cell homeostasis, activation, and antitumor function. J Immunol.

[59] Nicolai CJ (2020). NK cells mediate clearance of CD8^+^ T cell-resistant tumors in response to STING agonists. Sci Immunol.

[60] Gautier G (2005). A type I interferon autocrine-paracrine loop is involved in Toll-like receptor-induced interleukin-12p70 secretion by dendritic cells. J Exp Med.

[61] Xu HC (2014). Type I interferon protects antiviral CD8+ T cells from NK cell cytotoxicity. Immunity.

[62] Shanker A (2010). Cooperative action of CD8 T lymphocytes and natural killer cells controls tumour growth under conditions of restricted T-cell receptor diversity. Immunology.

[63] Kohlhapp FJ (2015). NK cells and CD8+ T cells cooperate to improve therapeutic responses in melanoma treated with interleukin-2 (IL-2) and CTLA-4 blockade. J Immunother Cancer.

[64] Upadhyay R (2021). A critical role for Fas-mediated off-target tumor killing in T-cell immunotherapy. Cancer Discov.

[65] Lerner EC (2023). CD8^+^ T cells maintain killing of MHC-I-negative tumor cells through the NKG2D-NKG2DL axis. Nat Cancer.

[66] Zhang B (2008). IFN-gamma- and TNF-dependent bystander eradication of antigen-loss variants in established mouse cancers. J Clin Invest.

[67] Gutjahr A (2019). The STING ligand cGAMP potentiates the efficacy of vaccine-induced CD8+ T cells. JCI Insight.

[68] Demaria O (2015). STING activation of tumor endothelial cells initiates spontaneous and therapeutic antitumor immunity. Proc Natl Acad Sci U S A.

[69] Zhu Y (2019). STING: a master regulator in the cancer-immunity cycle. Mol Cancer.

[70] Larkin B (2017). Cutting edge: activation of STING in T cells induces type I IFN responses and cell death. J Immunol.

[71] Hines JB (2023). The development of STING agonists and emerging results as a cancer immunotherapy. Curr Oncol Rep.

[72] Kraman M (2010). Suppression of antitumor immunity by stromal cells expressing fibroblast activation protein-alpha. Science.

[73] Kosti P (2021). Generation of hypoxia-sensing chimeric antigen receptor T cells. STAR Protoc.

[74] Risso D (2014). Normalization of RNA-seq data using factor analysis of control genes or samples. Nat Biotechnol.

[75] Love MI (2014). Moderated estimation of fold change and dispersion for RNA-seq data with DESeq2. Genome Biol.

[76] Raudvere U (2019). g:Profiler: a web server for functional enrichment analysis and conversions of gene lists (2019 update). Nucleic Acids Res.

[77] Elso CM (2004). Leishmaniasis host response loci (lmr1-3) modify disease severity through a Th1/Th2-independent pathway. Genes Immun.

