# Supplementary material for: Tumor microenvironments with an active type I IFN response are sensitive to inhibitors of heme degradation
Source: JCI Insight. 2025 Jul 8;10(16):e191017. doi: 10.1172/jci.insight.191017 (PMC12406722; doi:10.1172/jci.insight.191017)
Supplement: Supplemental data [file jciinsight-10-191017-s143.pdf]

Supplemental Material

Tumor microenvironments with an active type-I interferon response are sensitive to inhibitors of heme degradation

Dominika Sosnowska, Tik Shing Cheung, Jit Sarkar, James W. Opzoomer, Karen T. Feehan, Joanne E. Anstee, Chloé A. Woodman, Mohamed Reda Keddar, Kalum Clayton, Samira Ali, William Macmorland, Dorothy D. Yang, James Rosekilly, Cheryl E. Gillett, Francesca D. Ciccarelli, Richard Buus, James Spicer, Anita Grigoriadis and James N. Arnold

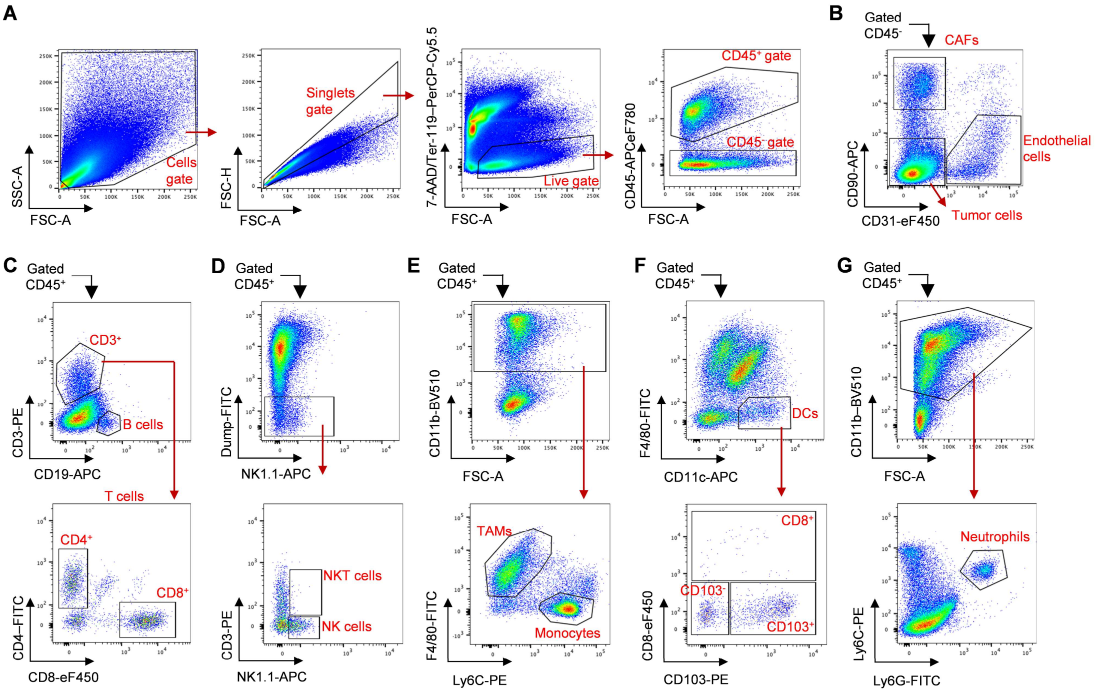

Supplemental Figure 1. Flow cytometry gating strategy for stromal populations. Gating strategy for identifying stromal populations from enzyme-dispersed *MMTV-PyMT* tumors using flow cytometry. Positive gates are applied based upon FMO stains. **(A)** Representative gating strategy for identifying single, live (7AAD$^-$) nucleated (Ter119$^-$) stromal immune (CD45$^+$) and non-immune (CD45$^-$) populations in enzyme-dispersed tumors from *MMTV-PyMT* mice using flow cytometry. **(B-G)** subsequent gating from the live cell gate for non-immune (CD45$^-$) populations of CAFs (CD90$^+$CD31$^-$), endothelial cells (CD90$^-$CD31$^+$) and tumor cells (CD90$^-$CD31$^-$) **(B)**, and immune (CD45$^+$) populations including B-cells (CD19$^+$), CD4$^+$ T-cells (CD3$^+$CD4$^+$), CD8$^+$ T-cells (CD3$^+$CD8$^+$) **(C)**, NK (Dump$^-$NK1.1$^+$CD3$^-$) and NK T-cells (Dump$^-$NK1.1$^+$CD3$^+$) **(D)**, TAMs (CD11b$^+$F4/80$^+$) and monocytes (CD11b$^+$Ly6C$^+$F4/80$^{low/-}$) **(E)**, DCs (CD11c$^+$F4/80$^-$) and DC subsets based CD8 and CD103 expression **(F)** and neutrophils (CD11b$^+$Ly6C$^+$Ly6G$^+$) **(G)**. Dump channel includes the markers: CD4, CD8, CD19, Gr-1, F4/80, MHCII).

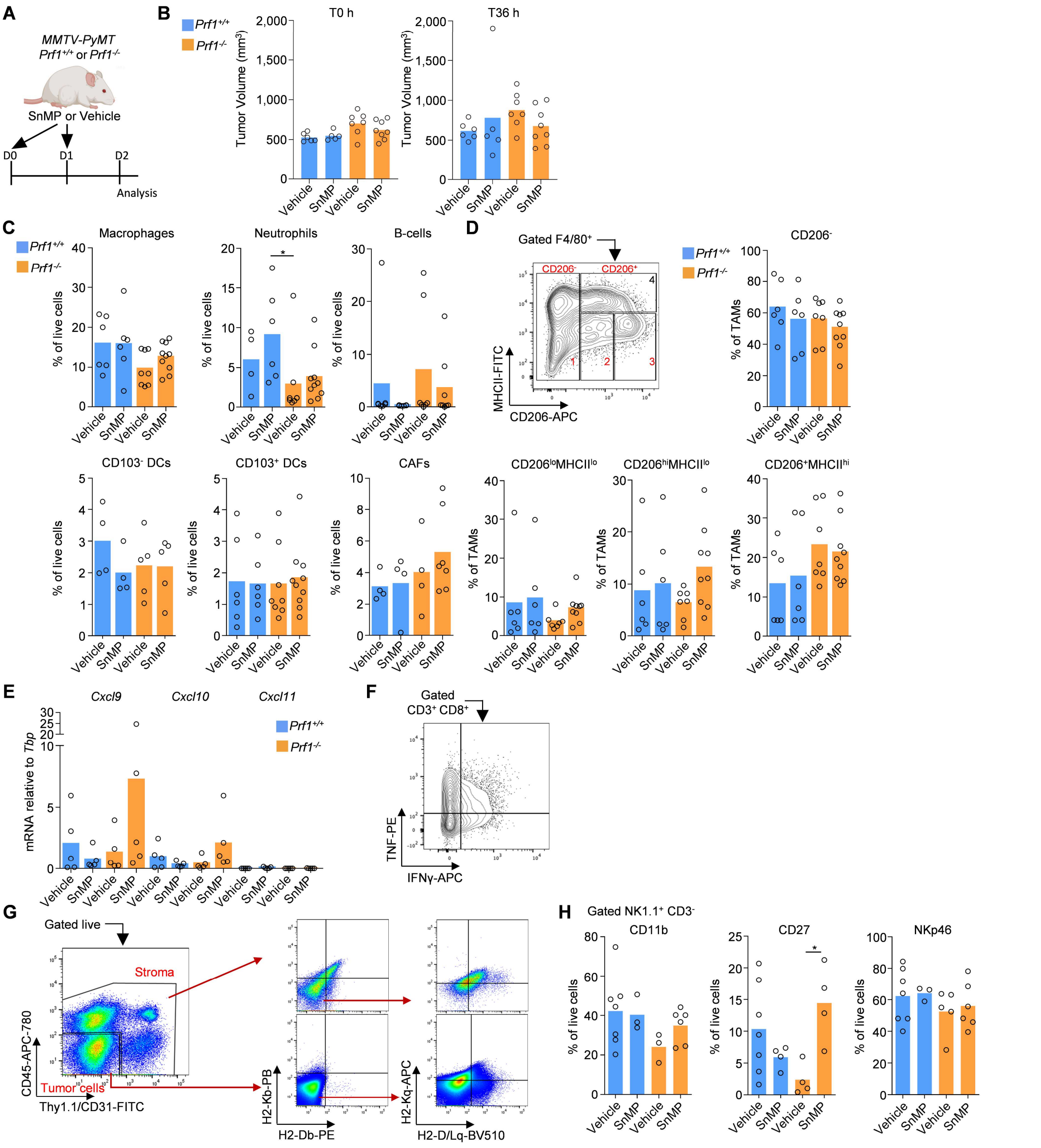

Supplemental Figure 2. Characterization of the TME of *Prf1*$^{+/+}$ or *Prf1*$^{-/-}$ *MMTV-PyMT* tumors and response to HO inhibition. (A-H) Schematic representing the acute dosing strategy for SnMP (25 μmol/kg daily) or respective vehicle in *Prf1*$^{+/+}$ or *Prf1*$^{-/-}$ *MMTV-PyMT* mice **(A)**. T0 h and T36 h tumor volumes **(B)**, at which point tumors were harvested, enzyme-dispersed and cell populations analyzed by flow cytometry for the frequency of indicated live stromal cells **(C)**, F4/80^{+} TAM subsets based on their respective expression of CD206 and MHCII **(D)** (cohorts of n=4-9 mice). mRNA was extracted from tumor tissue and probed for expression of *Cxcl9, Cxcl10, Cxcl11* relative to the house-keeping gene *Tbp* (cohorts of n=5 mice) **(E)**. Representative gating strategy for gated CD8^{+} T cell expression of TNF-α and IFN-γ **(F)** and gated live tumor and stroma expression of MHCI haplotypes **(G)**. Quantitation of gated NK cells and their surface expression of the indicated markers associated with NK cell activity and function **(H)** (cohorts of n=3-7 mice). Image in panel **(A)** was created using *BioRender* software. Bar charts show the mean and the dots show individual data points from individual tumors and mice. * *P*<0.05.

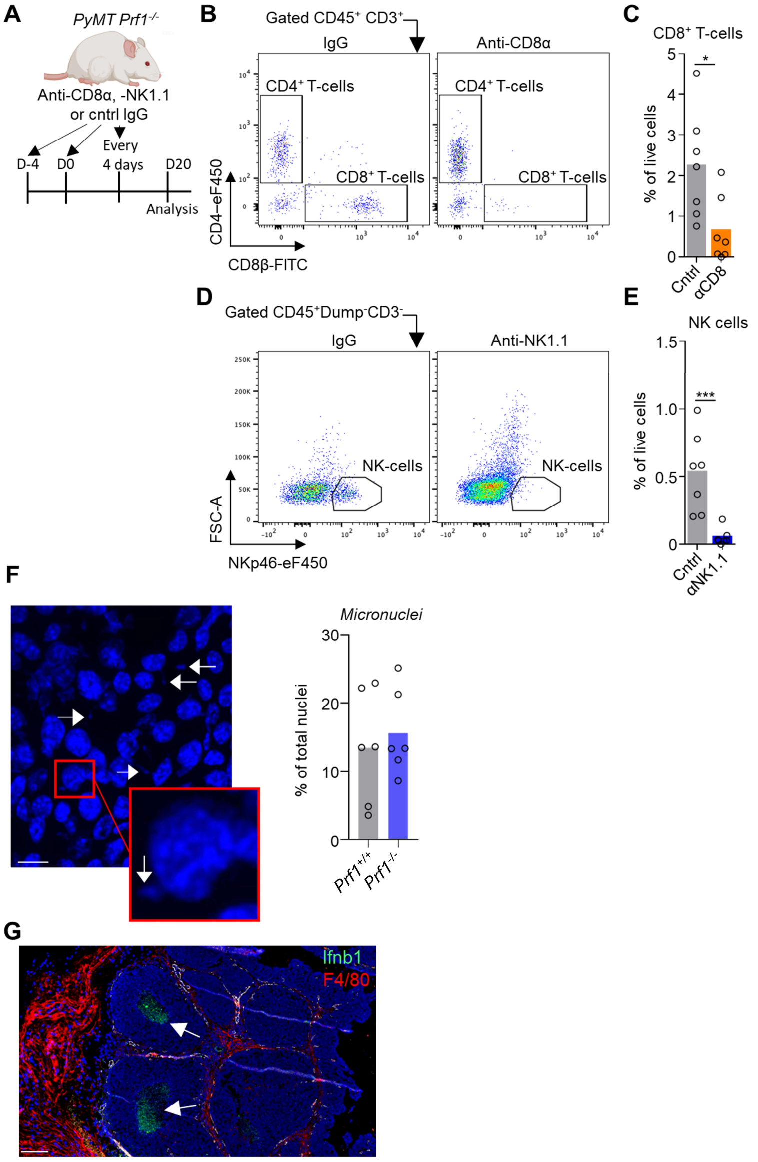

Supplemental Figure 3. Characterization of the TME of *MMTV-PyMT* tumors. **(A-E)** Schematic representing the dosing strategy for the immune-depleting anti-CD8α or anti-NK1.1 antibodies in *MMTV-PyMT* mice **(A)**. Flow cytometry gating strategy for tumor infiltrating CD8$^+$ (CD3$^+$ CD8β$^+$) and CD4$^+$ (CD3$^+$ CD4$^+$) T cells from a representative *MMTV-PyMT* mouse administered with or without immune-depleting anti-CD8α antibodies **(B)** and their quantification **(C)** or analysis of tumor infiltrating NK cells from a representative *MMTV-PyMT* mouse administered with or without immune-depleting anti-NK1.1 antibodies **(D)** and their quantification (n=7) **(E)**. **(F)** Representative image of a frozen section of *MMTV-PyMT* tumor stained with DAPI (nuclei; blue) (left panel) and quantitation (right) from *Prf1*$^{+/+}$ (n=6) or *Prf1*$^{-/-}$ (n=6) mice. Arrows point to examples of micronuclei. Scale bar represents 10 µm. **(G)** Representative image of an FFPE tumor section from a *Prf1*$^{-/-}$ *MMTV-PyMT* mouse tumor stained with; DAPI (nuclei; blue), antibodies against F4/80 (red) and probed for *Ifnb1* mRNA (green). Scale bar represents 100 µm. Arrow points to the region of central necrosis. Image in panel **(A)** was created using *BioRender* software. The dump channel used in **(D)** contains the marker CD4, CD8, CD19, Gr-1, F4/80, MHCII. Bar charts show the mean and the dots show individual data points from individual tumors and mice. * P<0.05, *** P<0.001.

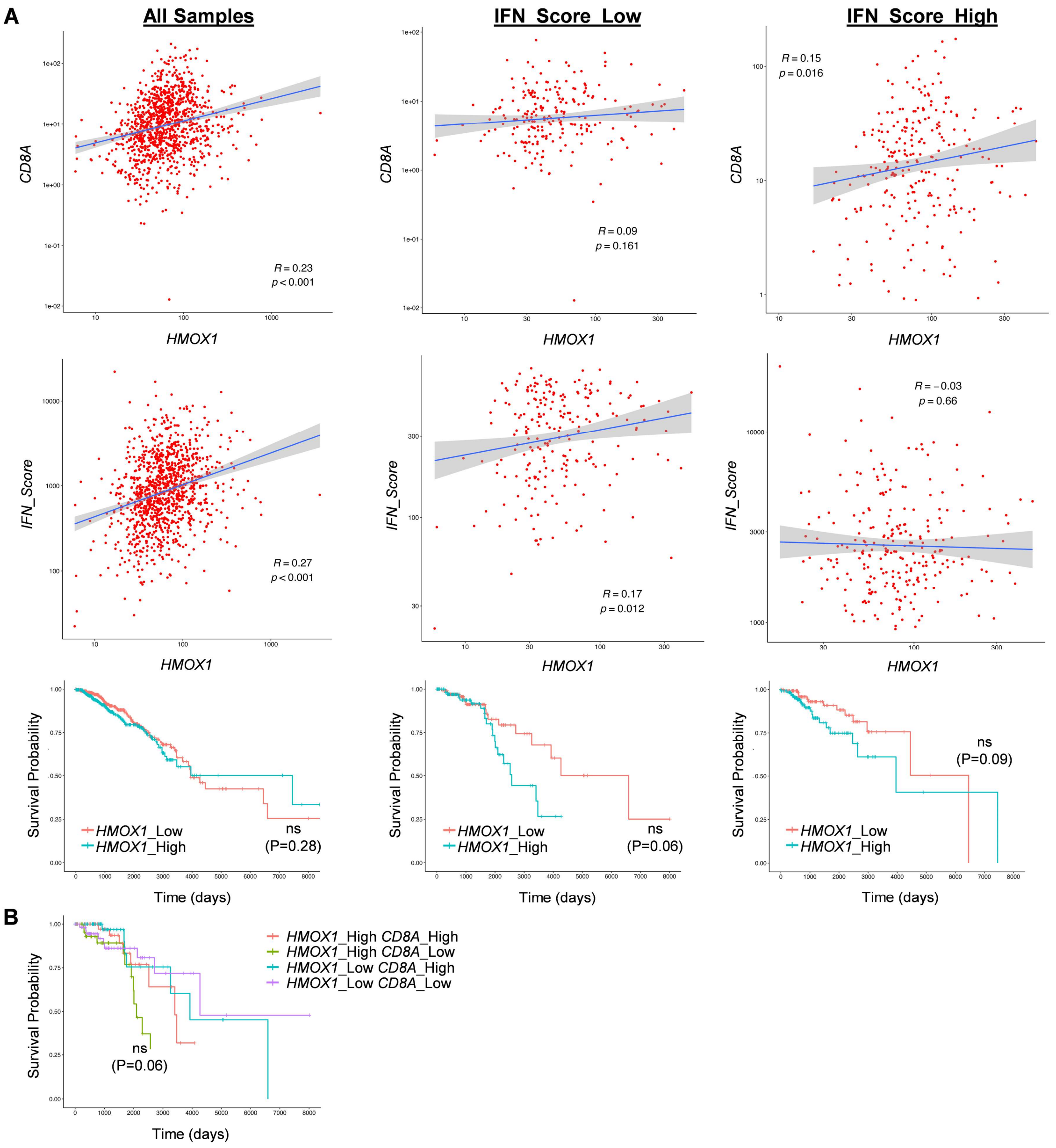

Supplemental Figure 4. **Correlation analyses and survival curves for human breast cancer subgroups for type-I IFN signature expressors (A)** Pearson's correlation analysis for *HMOX1* expression with *CD8A* expression (top panels) and IFN Score (middle panels) (obtained by summing up *BST2, IFI44, ISG15, IFNB1* expression) for all patients (n=1083), low IFN Score expressors (n=225, low expression of all four signature genes *BST2, IFI44, ISG15, IFNB1*) and high IFN Score expressors (n=253, high expression of all four signature genes *BST2, IFI44, ISG15, IFNB1*). Survival analyses for *HMOX1*-High and *HMOX1*-Low sub-groups across all aforesaid 3 groups (bottom panels). **(B)** Human breast cancer patients (n=225) were selected for low expression of all four signature genes (*BST2, IFI44, ISG15, IFNB1*) generated using the TCGA Breast Cancer data. Subgroups contained *HMOX1*-High-*CD8A*-High (n=61), *HMOX1*-High-*CD8A*-Low (n=51), *HMOX1*-Low-*CD8A*-High (n=51), *HMOX1*-Low-*CD8A*-Low (n=62) patients. Lines display the mean, ns- non-significant.

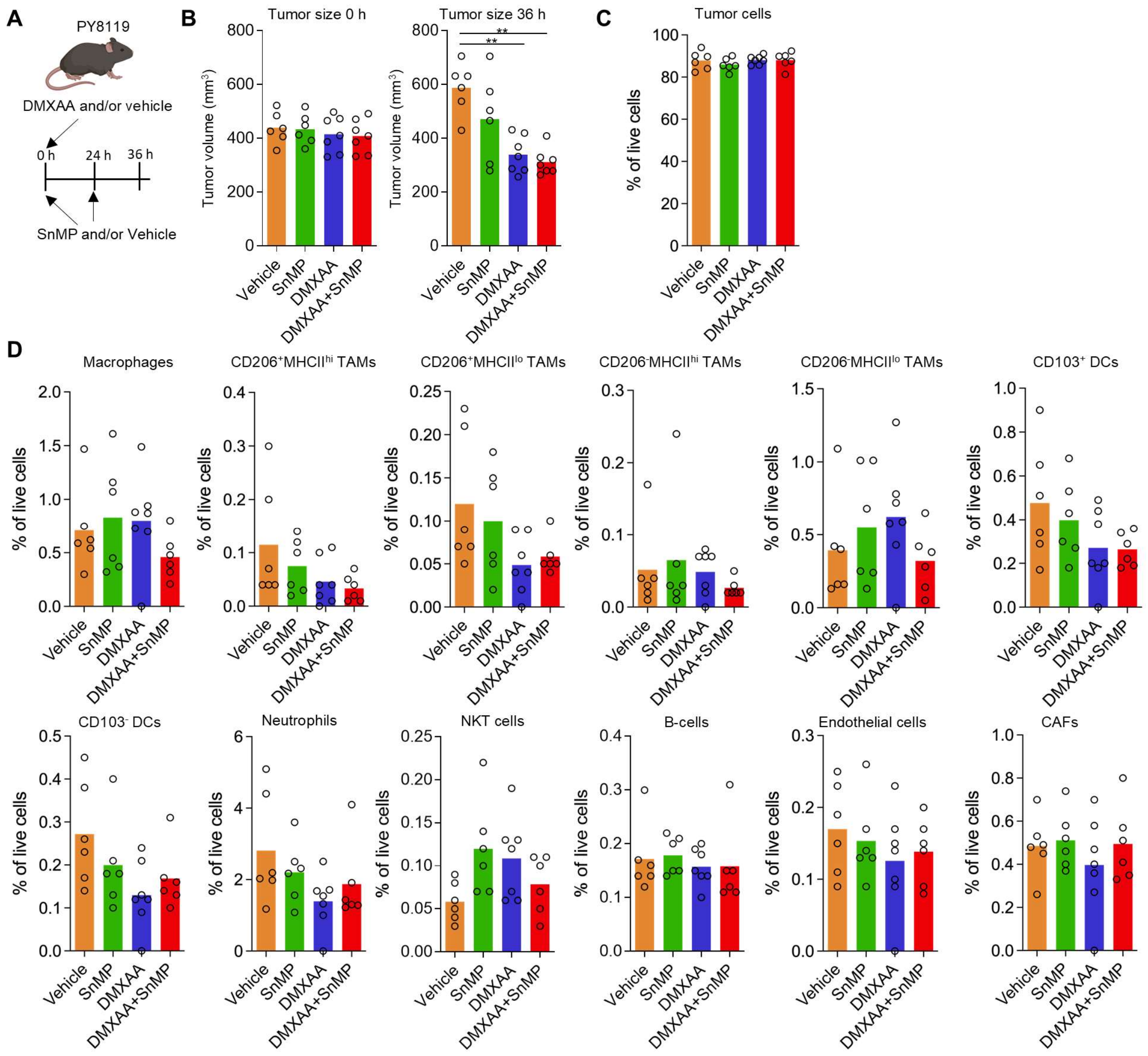

Supplemental Figure 5. Characterization of the TME of PY8119 tumors treated with DMXAA and SnMP. (A-D) Schematic representing the acute dosing strategy for SnMP and/or DMXAA and/or respective vehicles in mice bearing established PY8119 tumors **(A)**. Tumor volumes were taken at the beginning and at 36 h post initiation of treatment **(B)** at which point tumors were harvested, enzyme-dispersed and cell populations analyzed by flow cytometry for the frequency of live tumor cells **(C)** and indicated stromal populations **(D)** (cohorts of n=6-7 mice). Image in panel **(A)** was created using *BioRender* software. Bar charts show the mean and the dots show individual data points from individual tumors and mice. ** *P*<0.01.
